# Supplementary material for: Contrasting the potential benefits of early invasive coronary angiography in acute and chronic myocardial injury patterns
Source: PLoS One. 2023 Jun 15;18(6):e0286157. doi: 10.1371/journal.pone.0286157 (PMC10270641; doi:10.1371/journal.pone.0286157)
Supplement: S1 File — (DOCX) [file pone.0286157.s001.docx]

**Supporting Information**

*Inverse probability treatment weighting for Invasive Coronary Angiography*

To attempt to account for differences in patients referred and not referred for invasive coronary angiography (ICA), and logistic regression model with ICA as the dependent variable, the following independent variables were used: age <55 years (continuous), gender, diabetes, hypertension peak observed troponin within 12 hours, troponin pattern, prior MI, prior heart failure, prior CVA, prior PCI, prior CABG, prior atrial fibrillation and known COPD. The predicted probability of receipt of ICA was then calculated, and patients with a probability of less than 1% and those greater than 92.5% trimmed. Balance of final weight population is displayed in the following density plot (S1 Fig), and assessed by differences in the standardized differential (S1 Table).

Persistent imbalance of the characteristics of prior MI and diabetes is noted. These were included in the logistic regression models exploring the relationship between ICA and 12-month death or MI.

**S1 Table. Balance of final weight population assessed by standardized differential differences.**

|  | Mean in treated | Mean in untreated | Standardized differential |
| --- | --- | --- | --- |
| Age | 64.35 | 64.96 | -0.046 |
| Gender | 0.37 | 0.39 | -0.028 |
| Hypertension | 0.44 | 0.39 | 0.087 |
| Diabetes | 0.26 | 0.22 | 0.111 |
| Prior acute myocardial infarction | 0.17 | 0.13 | 0.109 |
| Prior heart failure | 0.06 | 0.07 | -0.050 |
| Prior atrial fibrillation | 0.13 | 0.12 | 0.038 |
| Prior chronic obstructive pulmonary disease | 0.07 | 0.08 | -0.024 |
| Prior cerebrovascular accident | 0.03 | 0.03 | -0.002 |
| Prior PCI | 0.14 | 0.12 | 0.061 |
| Prior CABG | 0.06 | 0.04 | 0.097 |
| eGFR | 76.01 | 76.94 | -0.043 |
| Peak hs-cTnT level | 34.92 | 33.72 | 0.007 |
| Abbreviations: PCI = percutaneous intervention; CABG = coronary artery bypass grafting; eGFR = estimated glomerular filtration rate (ml/min/1.73m^2^); hs-cTnT = high sensitivity cardiac troponin T | | | |

**S1 Fig. Density plot of balance of final weight population**
