# Supplementary material for: Contrasting the potential benefits of early invasive coronary angiography in acute and chronic myocardial injury patterns
Source: PLoS One. 2023 Jun 15;18(6):e0286157. doi: 10.1371/journal.pone.0286157 (PMC10270641; doi:10.1371/journal.pone.0286157)
Supplement: Attachment — Submitted filename: Response to reviewers.docx [file pone.0286157.s003.docx]

# Reviewers’ comments

***Comment 1***

**You address an important clinical question particularly in the setting of troponin elevation perioperatively in non cardiac surgery.**

**The majority of the troponin elevations are assumed to be due to type 2 MIs and are deemed not to require ICA.**

**What the reader needs from your paper is clear indications when to require ICA in the absence of clear ECG changes. Should they rely on the extent of the troponin elevation or should they require any additional information such as wall motion abnormalities in individuals who had a baseline echo that did not reveal any. Or other clear indications on who might benefit and in whom it would be a waste of resources without any benefit to the patient.**

***Response 1***

We thank the reviewer for the comment, and recognize the clinical challenges in differentiating Type 2 MI from Type 1 MI based on clinical presentation, and even ECG criteria or wall motion abnormalities on non-invasive imaging.

In this analysis, the majority of acute injury cases were Type 1 MI.  Importantly, since their differentiation depend on clarity over the pathophysiologic process underlying the myocardial injury (supply/demand ischaemia versus plaque rupture), some may consider assessment of the coronary anatomy/pathology as essential in the diagnostic process.

This analysis takes a different approach. By exploring the profile and peak concentration of troponin T as a determinant of “prognostic benefit” from invasive coronary angiography (as opposed to the use in diagnosis), we have suggested that there is are threshold values, depending on the profile, below which a benefit from an invasive coronary is not apparent, and therefore alternative strategies for coronary investigation, such as CT coronary angiography, may also be a viable strategy. Hence, this analysis suggests that in patients with an acute myocardial injury pattern, regardless of whether the diagnosis is Type 1 MI, Type 2 MI or acute injury, the early invasive strategy among patients with a peak troponin T less than ~100ng/L is unlikely to impact future death or MI. This insight may help clinicians consider alternative investigative approaches to those patients with low level troponin elevations.
