# Supplementary material for: Contrasting the potential benefits of early invasive coronary angiography in acute and chronic myocardial injury patterns
Source: PLoS One. 2023 Jun 15;18(6):e0286157. doi: 10.1371/journal.pone.0286157 (PMC10270641; doi:10.1371/journal.pone.0286157)
Supplement: Attachment — Submitted filename: Response to reviewers 09.03.2023.docx [file pone.0286157.s004.docx]

**Response to reviewer comments**

1. **In figure 1 I do not understand who is inserted in the third group (acute MI??).**

There were 3 groups of patients included in this study.

Patients in the “non-elevated troponin group” were defined as patients with serial troponins between 3-14ng/L.

Patients in the “acute myocardial injury group” (or hs-cTnT elevation with dynamic change) were defined as patients with at least 1 troponin result > 14ng/L and serial levels associated with rise and/or fall of > 20%. This group was further subclassified into Type 1 MI, Type 2 MI, or acute myocardial injury, utilising the 4^th^ Universal Definition of MI definitions.

Patients in the “chronic myocardial injury group” (or non-dynamic hs-cTnT elevation) were defined as patients with at least 1 hs-cTnT result > 14ng/L but serial levels were associated with < 20% overall change.

Figure 1 has been amended for clarity.

2. **In table II angiography has been performed in 182 patients. Overall coronary revascularisation was reported in 83 (45.6%) but thereafter are described PCI 20 (11.0%) and CABG 102 (56.0%) - a total of 122 patients. The numbers are not concordant.**

We thank the reviewer for the question. There is a transcription error in the table that has now been corrected. Among those with a dynamic pattern, 102 received some form of revasculrisation. Of the 102, 83 patients received PCI, whilst 20 received CABG – 1 person received both PCI and CABG.

3. **Whether patients in RAPID study were included without ECG examination how was diagnosis of type of MI made ? The patients from the two study had ED evaluation for chest pain ? It should be reported in the description of the study.**

The design of RAPID-TnT has previously been published - <https://www.sciencedirect.com/science/article/pii/S000287031730145X?casa_token=YJrw8RmRTrUAAAAA:0Xj_t2GLiBT0W2F-TokR042F_WlmyhDb8VLdBZxCQUtxCSMhI-QeI9Did0x_cBhyLcd_tEdeNIQ>.

The inclusion criteria for patients in RAPID TnT were:

- Clinical features of chest pain or suspected ACS as the principal cause for investigation
- Baseline ECG interpreted as not definitive for coronary ischaemia
- Age ≥ 18yo
- Willing to give written informed consent

Patients were then randomised to the 0/1-hour protocol, with the result to determine the patient’s subsequent care. There were 3 possible outcomes:

1. Baseline troponin < 5, or <12ng/L and change in troponin over 1 hour < 3ng/L -> rule-out i.e. discharge to primary care
2. Baseline troponin ≥ 52ng/L, or change over 1 hour of ≥ 5ng/L -> rule-in i.e. admit to hospital for management of MI
3. Baseline troponin 13-51ng/L and a change <5ng/L over 1 hour, or baseline troponin < 12ng/L and a change of 3-4ng/L over 1 hour -> observe

The design of hs-cTnT has previously been published here. <https://www.ahajournals.org/doi/10.1161/CIRCOUTCOMES.115.002488>.

Patients presenting to emergency departments with clinical features or suspected ACS (chest pain or overwhelming shortness of breath > 10 minutes at rest < 24 hours from the time of presentation) in whom the treating physician deemed it necessary to measure serum troponin were eligible.

4. **The number of patients with chronic myocardial injury underwent angiography was close to 15% of the whole population and only a small number were studied at 12 months with 3.7% of further revascularization Overall mortality was not different from the other group , the other end points are differently reported in table 2 and 3 ( for example ACS death in table 2 is not reported in table 3, Death, MI, and unstable angina in table 3 ) . A table comparing the two groups may be useful.**

We apologise for the confusion. There is a transcription error in the table that has now been corrected and the labels for the clinical outcome have also been corrected. Tables 2 and 3 were intended to represent exactly the same parameters.

5. **The paper is long and discussion not easy to follow. Please simplify for the reader.**

We have reviewed the manuscript and have made amendments to enhance clarity.
